# The Mechanobiological Hypothesis of Piezo Family-Mediated Exercise Intervention in Spinal Cord Injury Recovery

**DOI:** 10.1155/np/8868688

**Published:** 2025-11-14

**Authors:** Chenyu Li, Yuping Wang, Qianxi Li, Xinyan Li, Jinghua Qian, Xuemei Li, Xin Zhang

**Affiliations:** ^1^School of Sports Medicine and Rehabilitation, Beijing Sport University, Beijing, China; ^2^School of Nursing, Jiamusi Vocational College, Jiamusi, China

## Abstract

Exercise training plays a pivotal role in neural repair and secondary injury prevention following spinal cord injury (SCI) and is widely implemented in clinical rehabilitation. It induces adaptive changes and remodeling within the nervous system of SCI patients, thereby improving functional impairments. The mechanisms underlying this adaptive remodeling involve complex signaling pathways, with mechanosensation and mechanotransduction being indispensable. Mechanosensitive ion channels (such as Piezo channels) sense and transduce mechanical forces generated during exercise, triggering downstream biochemical reactions that regulate cellular functions and ultimately promote functional recovery. This review systematically synthesizes evidence from Piezo channel-related animal studies and clinical research, focusing on their role in reshaping the structure and function of the nervous system through exercise intervention post-SCI. The study aims to elucidate the molecular mechanisms by which Piezo channels mediate exercise-induced functional recovery after SCI, providing a theoretical foundation for developing precision exercise prescriptions to facilitate functional reconstruction and rehabilitation in patients.

## 1. Introduction

The incidence of spinal cord injury (SCI) shows a persistent upward trend globally. In China—the world's most populous nation—the incidence of traumatic SCI has reached 65.15 per million population [1], imposing a substantial burden on both individuals and society. Consequently, seeking effective interventions to improve SCI outcomes is crucial for enhancing population health and well-being [2].

Exercise training, as a cost-effective therapeutic approach, enhances patients' quality of life by stimulating neuroplasticity, preserving musculoskeletal function, preventing complications, and modulating immune responses. It also alleviates psychological distress and accelerates rehabilitation [3–5]. Personalizing exercise prescriptions requires understanding the patient's physiological responses, personal capabilities, goals, priorities, injury characteristics, and equipment availability [6]. Moreover, individual exercise responsiveness is modulated by genetic polymorphisms [7]. Currently, evidence-based approaches for optimizing post-SCI exercise regimens remain limited, which relates to methodological deficiencies in research approaches and the reliance on subjective metrics (e.g., rating of perceived exertion, RPE) for monitoring exercise intensity, alongside a critical shortage of objective biomarkers [8, 9]. This deficiency reflects fundamental gaps in mechanistic research on exercise-induced SCI recovery. Consequently, elucidating the mechanisms underlying exercise-mediated effects on SCI is of critical importance.

Exercise training is inextricably linked to mechanotransduction—a process critical for sensing neuronal migration, hemodynamic regulation, immune responses, and musculoskeletal tissue differentiation [10–14]. This biological process initiates when mechanosensors (including ion channels, transmembrane adhesion receptors, sarcomeric proteins, and cell-surface receptors) detect mechanical stimuli. These forces are subsequently converted into electrochemical signals, triggering adaptive cellular responses [15]. During exercise, energy expenditure generates both endogenous and exogenous mechanical forces that orchestrate tissue remodeling. Specifically, mechanical loading regulates peripheral nerve architecture formation and physiological function maintenance, modulates immune cell activity, and enhances skeletal muscle strengthening coupled with bone reconstruction [16–19]. The therapeutic benefits of mechanical stimulation fundamentally depend on mechanotransduction. Consequently, exercise-induced advantages may ultimately arise through the mechanotransduction-mediated conversion of mechanical forces into electrochemical signaling cascades.

Simultaneously, traumatic SCI—induced by mechanical forces—is characterized by disruption of neural parenchyma, vascular rupture, and destruction of axonal networks with concomitant compromise of the glial membrane. Injury severity varies significantly with the nature of mechanical stimulation (e.g., penetrating injuries elicit distinct pathological responses compared to tensile or compressive forces) [20, 21]. Micropipette aspiration techniques have quantitatively delineated the dynamic evolution of spinal cord tissue biomechanical properties post-SCI: By Day 3, tissues exhibit significant softening and enhanced viscoelasticity; baseline mechanical properties partially restore during Weeks 4–6; and by Week 12, fibrotic scarring with supraphysiological stiffness develops at the lesion epicenter [22]. Consequently, post-SCI alterations in spinal tissue mechanics are likely mediated through mechanotransduction pathways.

Furthermore, in the mechanotransduction process, the activation of mechanosensitive ion channels underpins numerous fundamental physiological processes requiring mechanical force sensing [23]. The discovery of the Piezo family—a novel class of mechanosensitive ion channels in mammals—has opened new avenues for investigating mechanotransduction's role in human health and disease [24]. Through siRNA knockdown and patch-clamp electrophysiology, PIEZO1 was first identified as the mediator of mechanically activated cationic currents in mouse neuroblastoma cells, while PIEZO2 was shown to regulate rapidly adapting mechanosensitive currents in sensory neurons, such as dorsal root ganglion neurons [25]. Piezo1 channels primarily transduce mechanical signals to govern diverse physiological responses, whereas Piezo2 channels are indispensable for tactile sensation, proprioception, and mechanical nociception [26]. Piezos exhibit profound links to motor function. Both Piezo1 and Piezo2 channels serve as key molecular transducers for transcranial focused ultrasound (tFUS) activation of the motor cortex, eliciting limb movement responses; their functional impairment significantly reduces response success rates and prolongs latency [27]. Interestingly, reports have found that Olympic medal-winning athletes carrying specific variants of the *PIEZO1* gene and the *SDC2* gene (which may modulate the interaction between PIEZO1 and PIEZO2) exhibit abnormal blood indicators and exceptional athletic performance potentially linked to “excessive mechanotransduction” resulting from long-term high-intensity training [28]. Elite athletes experience excessive mechanotransduction during high-intensity training and competition, with genetic variations in Piezo genes influencing their adaptive responses—factors reflected in their peak performance. Additionally, studies in rat soleus muscles demonstrate that exercise-induced mechanical stimulation activates Piezo channels to trigger afferent signaling, thereby mediating the mechanical component of the exercise pressor reflex. This results in elevated blood pressure, increased heart rate, and sympathetic activation [29]. Thus, Piezo channels represent a fundamental mechanistic pathway through which exercise exerts its physiological effects.

**Figure 1:**
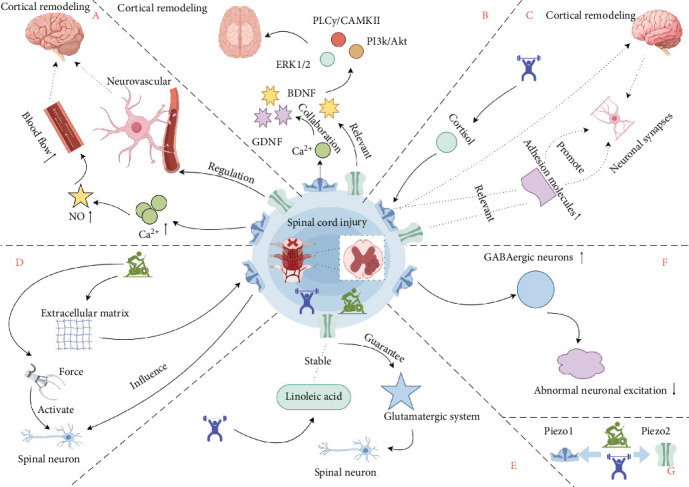
This schematic illustrates the system integrating the mechanisms by which Piezo channels mediate exercise training-induced neural remodeling after spinal cord injury. (A) Piezo channel opening promotes cortical remodeling through neurovascular coupling. (B) The synergistic effect of Piezo channels and neurotrophic factors promotes neural plasticity via key signaling pathways (PI3K/Akt, PLCγ/CAMKII, and ERK1/2). (C) Exercise influences Piezo channels via cortisol activation, and Piezo channels promote synaptic connections through adhesion molecules. (D) Exercise-induced changes in the mechanical properties of the extracellular matrix regulate spinal neurons (e.g., axon growth and migration) through Piezo channels. (E) Piezo2 function is associated with the stability of the glutamatergic system (VGLUT1/2). Exercise may stabilize Piezo2 by elevating linoleic acid levels, exerting neuroprotective effects. (F) This module indicates the regulatory relationship between abnormal excitability of GABAergic neurons and mechanosensitive channels Piezo1/2. (G) Exercise activates Piezo1/2. Image source: Figdraw.

Therefore, this paper proposes the core scientific hypothesis: the Piezo mechanosensitive ion channel family serves as a key molecular target mediating exercise intervention-induced neural repair and functional recovery after SCI (Figure 1). The following sections will focus on the critical pathophysiological changes within the central nervous system (specifically the cerebral cortex and spinal cord) post-SCI. It will discuss the potential mechanisms by which exercise interventions may promote neural regeneration, remodeling, and functional recovery through the regulation of Piezo channel activity, thereby outlining future research directions for the precision of exercise prescriptions.

## 2. Introduction to Piezo Channels

### 2.1. Piezo1

Piezo1 achieves mechanotransduction through its unique structure. High-resolution cryo-electron microscopy structures reveal that mouse Piezo1 forms a homotrimer composed of three subunits. The overall architecture adopts a distinctive three-bladed propeller-shaped conformation. Each monomer contains at least 26 transmembrane helices. The transmembrane domain exhibits distinct curvature and is organized into multiple Piezo repeat units. This structural feature may enable Piezo1 to sense mechanical forces by inducing localized changes in membrane curvature. Furthermore, it relies on allosteric conformational changes within its beam-anchor domains to transmit mechanical signals to the central ion-conducting pore, thereby converting membrane tension into electrical signals [30].

The core mechanosensory receptor function of Piezo1 is highly evolutionarily conserved. In the invertebrate *Drosophila melanogaster*, the Piezo1 homolog gene *pzl* has been identified. Loss of *pzl* disrupts the ability of chordotonal neurons to sense static body position changes, resulting in severe defects in locomotor patterns and postural control. Notably, this function can be rescued by cross-species expression of human or mouse Piezo1, confirming the evolutionary conservation of the Piezo family in animal motor control [31]. Beyond the animal kingdom, the *Arabidopsis thaliana* Piezo1 protein localizes to mechanosensitive regions of the root tip, functioning as a mechanosensitive ion channel. It is essential for roots to sense soil mechanical impedance, trigger calcium signaling, and penetrate compact growth substrates, further demonstrating the evolutionary conservation of Piezo channels in mechanosensation across both plants and animals [32].

Throughout evolution, the distinct mechanical environments resulting from species-specific locomotion modes and lifestyles have driven subtle variations or tissue-specific expression patterns of Piezo1. For instance, swimming activity in zebrafish larvae directly activates Piezo1 channels in arterial endothelial cells via mechanical force, triggering localized calcium signaling events. This indicates that their unique locomotor pattern shapes tissue-specific Piezo1 function (particularly within the arterial system), rather than uniform whole-body expression [33]. Humans have also evolved distinct Piezo1 phenotypes (Table 1). In populations of African descent, the Piezo1 E756del mutation—an evolutionary trade-off selected for malaria resistance (achieved through a red blood cell dehydration mechanism that counters Plasmodium infection)—exhibits a gain-of-function effect. This effect progressively suppresses hepcidin with age, elevating the risk of iron overload [43]. Genetic sequencing has confirmed that dehydrated hereditary stomatocytosis (DHS) is driven by missense mutations within the C-terminal half of the PIEZO1 gene. The gain-of-function effect arises from delayed inactivation of the mechanically activated channel, ultimately leading to increased cation influx and cellular dehydration in red blood cells [44]. Such evolutionarily acquired mutations often entail physiological imbalances. However, under the selective pressures of specific African environments, populations of African descent have developed a phenotypic advantage in explosive power. Studies of Jamaican sprint athlete cohorts reveal a significantly higher prevalence of the PIEZO1 E756del gain-of-function mutation in their tendons compared to non-athlete controls, providing molecular evidence for adaptive evolution in human athletic performance [45]. Furthermore, reports suggest that prolonged excessive mechanotransduction in Olympic medal-winning athletes correlates with Piezo1 variants linked to blood parameter abnormalities, potentially signifying enhanced athletic capacity [28]. Therefore, mechanical stimuli generated by exercise may activate Piezo1, inducing adaptive physiological changes.

These studies on human polymorphisms reveal the pivotal role of PIEZO1 across multiple physiological systems, including the skeletal, sensory, hematological (blood), and vascular systems. Its genetic variations are associated with diverse disease phenotypes and physiological traits, underscoring the critical importance of PIEZO1 function for human health.

### 2.2. Piezo2

Both Piezo2 and Piezo1 are homotrimers with overall similar architectures. However, Piezo2 possesses a more complete N-terminal structure and a smaller radius of curvature. Its activation requires a higher mechanical force threshold and it is predominantly enriched in sensory neurons, serving as a key determinant of human mechanosensation [25, 46, 47]. Additionally, Piezo1 can act as a high-pass, band-pass, or low-pass filter depending on the stimulus waveform, owing to its inactivation kinetics. In contrast, Piezo2 consistently behaves as a low-pass filter under square-wave stimulation. Both channels rely on inactivation for frequency selectivity, but their filtering characteristics are regulated by channel-specific biophysical properties [48]. This demonstrates a degree of complementarity between the two channels. The evolutionary process of Piezo2 is also linked to the unique environments of species. For instance, compared to chickens, ducks—adapted for the special demands of underwater tactile foraging—have evolved a trigeminal nervous system with significantly higher Piezo2 expression and unique slow inactivation kinetics. This enables efficient conversion of sustained tactile stimuli into neural signals [49]. Piezo2 also exhibits exercise adaptability; its expression level in mouse knee joint cartilage significantly increases after exercise compared to sedentary groups [50].

## 3. Unleashing Repair Potential: The Hypothesis that Exercise Promotes Neural Regeneration After SCI by Modulating Piezo Channels

### 3.1. Exercise Modulates Remodeling of the Cortical Neural Circuitry via Piezo1

A substantial body of evidence indicates that exercise training following SCI may influence the plasticity of neural circuits in the cerebral cortex. In mouse models of incomplete SCI [51], exercise training promotes the reorganization of neural circuits and remodeling of the cerebral cortex through sensory input from muscle spindles. In studies observing exercise training initiated 1 week post-SCI in mice [52], 3 weeks of training demonstrated plasticity in the electrophysiological properties of neuronal cell membranes, while 6 weeks of training positively influenced changes in synaptic drive and increased synaptic connection strength. Furthermore, clinical studies have confirmed [53] that body-machine interface-based exercise training enhances the function of remaining viable nerves and muscles in SCI patients, also supporting plasticity in the cerebral cortex. This evidence collectively demonstrates the impact of exercise training on cortical plasticity after SCI.

Exercise training may influence post-SCI cortical plasticity through Piezo-mediated regulation of blood circulation. During incremental exercise, cerebral blood flow (CBF), cardiac output, and oxygen uptake increase simultaneously. This is likely due to shear stress-induced vasodilation and the increased demand for endothelium-derived nitric oxide (NO) to sustain this vasodilatory state [54]. Piezo1 plays a crucial role in sensing intravascular shear stress. Studies in mouse models show that Piezo1 activation leads to increased Ca^2+^ influx, subsequently triggering NO release and promoting vascular relaxation within the pulmonary circulation [55]. In studies on Alzheimer's disease mouse models, transcranial ultrasound stimulation and transcranial magneto-acoustic stimulation (involving magnetoelectric and mechanical forces) significantly increased hippocampal CBF and affected synaptic plasticity. However, this study did not include Piezo1 knockdown controls and did not establish a specific causal relationship between CBF and Piezo1. Nevertheless, observed concurrent changes in Piezo1 and CBF suggest a potential correlation [56]. Conversely, in a study on intracerebral hemorrhage, downregulation of Piezo1 using the mechanosensitive channel inhibitor GsMTx-4 restored CBF to normal levels [57]. Therefore, Piezo1 influences changes in cerebral blood volume within the cerebrovasculature, and its downregulation may reduce blood flow. Furthermore, Piezo2 plays a key role in mediating direct contact between specific neurons (e.g., Fam19a4/Nts-RGCs) and cerebral blood vessels, regulating the directional sprouting of penetrating vessels and the proper assembly of the three-dimensional vascular network. This mechanism exists in both the retina and cerebellum and is essential for maintaining cerebral microcirculatory function and ischemic tolerance [58]. This indicates that Piezo2 may also participate in regulating neurovascular unit function, although its specific role in exercise-mediated regulation of CBF and neurovascular coupling remains to be further investigated.

Exercise training may influence post-SCI cortical plasticity through Piezo-mediated regulation of Ca^2+^ signaling and the expression of brain-derived neurotrophic factor (BDNF) and glial cell-derived neurotrophic factor (GDNF). In animal models of endurance training, BDNF and GDNF influence cell survival and regeneration via the PI3K/Akt and ERK1/2 signaling pathways, while BDNF triggers neural plasticity through the PLCγ/CaMKII signaling pathway [59]. In in vitro cellular studies [60], the C-terminus of the Piezo1 protein plays a crucial role in Ca^2+^ influx and the activation of the ERK1/2 signaling pathway. Concurrently, Ca^2+^ influx and CaM (calmodulin) binding regulate CaMKII, which may in turn modulate PLCγ phosphorylation, impacting the PLCγ/CaMKII signaling pathway. Ca^2+^ also participates in TrkB signaling transduction, promoting neural plasticity and neural network formation [60, 61]. In studies across various systemic diseases [62–64], Piezo1 upregulation may partially activate the PI3K/AKT/mTOR pathway, while Piezo1 downregulation significantly suppresses the increase in Ca^2+^ signaling, thereby inhibiting mTOR phosphorylation. Notably, exercise, through activating the mTOR pathway, is crucial for processes, such as spine formation, neuronal activation, and axonal myelination [65]. Piezo2 acts as a negative regulator of BDNF release in differentiating Schwann cells; its loss of function leads to increased BDNF secretion via the TRPV4 channel [66]. This suggests that Piezo channel regulation of neurotrophic factor expression is cell-type specific, and its role within the cerebral cortex remains unclear. However, Piezo1 and Piezo2 act synergistically to confer high mechanosensitivity to tissues [67]. Therefore, Piezo2, acting as a mechanosensitive channel, may cooperate with Piezo1 or other channels to collectively participate in regulating neuronal function and neural remodeling processes, potentially through modulating Ca^2+^ signaling or other mechanisms.

Exercise training may promote cortical plasticity after SCI by modulating synaptic adhesion molecules to influence Piezo channels. Research indicates that, through functional screening using calcium imaging and electrophysiology, the adhesion molecule CADM1/SynCAM was found to slow the inactivation kinetics of Piezo1. Knockdown of the adhesion molecule gene accelerated Piezo1 inactivation [68]. Furthermore, in non-neuronal systems, Piezo2 is functionally linked to the cell adhesion molecule CDON via regulation of the Hedgehog signaling pathway: its low expression leads to downregulation of the CDON adhesion molecule in breast cancer, collectively promoting malignant tumor progression and poor prognosis [69]. Downregulation of adhesion molecules leads to synapse elimination [70]. Given that adhesion molecules (like CADM1) can regulate Piezo1 channel kinetics and their downregulation is associated with synapse elimination, this suggests adhesion molecules may indirectly participate in regulating synapse stability by influencing Piezo channel activity. However, a direct causal link between changes in Piezo channel activity and synapse elimination still requires experimental validation. Sustained aerobic exercise has been shown to enhance synaptic plasticity and function in the frontal cortex through selective splicing of neuropeptides, a family of synaptic adhesive molecules [71]. Therefore, exercise training may promote synaptic plasticity by modulating the expression or function of synaptic adhesion molecules, thereby influencing Piezo channel activity, and ultimately facilitating synaptic plasticity.

Furthermore, Piezo1 may influence post-SCI cortical plasticity through neuroendocrine changes induced by high-intensity exercise. Neurotrophic factors and the mTOR pathway exhibit intensity dependence within the motor cortex: both moderate and high-intensity treadmill training elevate BDNF expression and enhance mTOR pathway activity, whereas excessively high exercise intensity may negatively impact exercise endurance and survival [72]. In another study in the adult rat hippocampus, high-intensity exercise reduced expression of the neural plasticity marker GAP-43 but increased CAP-1 expression [73]. This indicates that exercise training influences cortical plasticity in an intensity-dependent manner. Cortisol is a key molecule inducing a decline in neural plasticity, and high-intensity exercise may negatively impact neuroplasticity due to elevated cortisol levels [74, 75]. Cortisol, as a glucocorticoid, acts similarly to dexamethasone. Studies show that high-dose dexamethasone can activate Piezo1 in macrophages, leading to Ca^2+^ influx and cytoskeletal remodeling [73, 76]. Concurrently, Piezo1 upregulation has been shown to be beneficial for synaptic plasticity in astrocytes and microglia [56, 77]. This raises a hypothesis worthy of investigation: could the cortisol elevation induced by high-intensity exercise potentially activate Piezo1 channels within the central nervous system (e.g., in neurons or glial cells) via a similar mechanism? If true, the ultimate effect of such activation on neural plasticity (promoting or inhibiting) under conditions of elevated cortisol would be complex and likely cell-type dependent. Whether and how Piezo1 directly senses or mediates the biological effects of different exercise intensities remains an important open scientific question.

### 3.2. Exercise Modulates the Structure and Function of Spinal Cord Neurons via Piezo1

Changes in the elastic modulus of the perineuronal matrix after exercise may mediate axonal growth and migration following SCI through Piezo1. In studies of *Xenopus* retinal ganglion cells [78], axons could transduce signals about the stiffness of the surrounding matrix via Piezo1. They grew faster, straighter, and more parallel on stiffer substrates, while exhibiting slower, exploratory growth with increased dispersion on softer substrates. Furthermore, in regions with significant stiffness gradients, Piezo1 directed axonal growth towards softer areas. Measurements of mechanical properties within the brain using magnetic resonance elastography after high-intensity interval training showed a significant decrease in brain stiffness [79]. Additionally, studies in animal models (mice and pigs) [80, 81] demonstrated that exercise training can affect the elastic modulus of both the subendothelial vascular matrix and the perivascular matrix. Concurrently, exercise training may increase the proliferation of ependymal cells, neural stem cells, and glial cells after SCI, thereby increasing cell density around the lesion site to promote neural repair [82–84]. Cell density is one factor influencing the elastic modulus of the surrounding matrix post-SCI [85]. Therefore, exercise might influence the signaling of the Piezo1 channel on the neuronal surface by modulating the elastic modulus of the perineuronal matrix, consequently affecting axonal growth and migration after SCI.

Mechanical forces generated by exercise and acting on neurons may mediate post-SCI neural growth, differentiation, and migration through Piezo1. Peripheral nerve-directed stretch can reduce neural stiffness [86], indicating that stress generated by exercise training can directly influence nerves. In in vitro studies using mouse NSC-34 and 50B11 cell lines [87], neural dynamics therapy might promote cell differentiation, neurite outgrowth, and neuronal survival by applying selective uniaxial repetitive tension to stimulate Piezo1 on nerves. Mechanical stimulation can also activate YAP and TAZ, transcriptional co-activators of the Hippo pathway, which are crucial for regulating the central nervous system. Activation of Piezo1 facilitates the nuclear translocation of YAP and TAZ within cells, thereby influencing the properties of neurons and glial cells [88]. Concurrently, YAP–TEAD interaction is necessary for promoting serum-induced astrogliogenesis in human neural stem cells, although how Piezo1-mediated Ca^2+^ signal transduction participates in regulating YAP translocation remains unclear [89]. Additionally, studies in human brain oligodendrocytes [90] suggest that Piezo1 may also play a role in regulating the proliferation, migration, and maturation of oligodendrocytes. Therefore, exercise training may influence post-SCI neuronal growth, differentiation, and migration through Piezo1.

Exercise training may also indirectly regulate Piezo2 function by influencing lipid metabolism, potentially mitigating neural injury. Piezo2 channel dysfunction impairs its mediated proton-based ultrafast signaling system: VGLUT1 disruption leads to a loss of protonated proprioceptive feedback to motor neurons, while VGLUT2 disruption disrupts hippocampal proton oscillation synchronization. This impairment triggers progressive motor neuron death, neuromuscular junction degeneration, and hippocampal dysfunction [91]. Notably, linoleic acid directly stabilizes Piezo2 protein–lipid interactions through its strong lipophilicity, blocking the TMEM120A/MyoD dissociation-induced proton leak pathway, thereby reversing acquired Piezo2 channelopathy-associated neurodegenerative injury [91]. Interestingly, exercise training significantly increases linoleic acid levels in skeletal muscle (plantaris) and liver [92]. This suggests a potential mechanism: exercise-induced elevation of linoleic acid levels might help stabilize Piezo2 function, maintain normal proton signaling, and consequently protect neurons from degenerative injury. However, the applicability of this hypothesis in the SCI context still requires experimental validation, including confirming whether post-SCI exercise effectively elevates linoleic acid levels in the central nervous system and whether these levels are sufficient to produce neuroprotective effects. Additionally, after SCI, exercise training specifically promotes increased VGLUT1^+^ innervation density in the rostral gray matter of the SCI zone, indicating its mediation of neural plasticity through enhanced glutamatergic synaptic transmission [93]. Furthermore, glutamatergic neurons in the lateral hypothalamic area drive post-SCI walking recovery via their VGLUT2-mediated neural projections; deep brain stimulation activating this pathway immediately improves walking ability and induces long-lasting neural remodeling [94]. Although the relationship between VGLUTs (primarily involved in glutamate transmission) and Piezo2-mediated proton signaling is not fully understood, these findings collectively highlight the importance of the glutamatergic system in exercise-promoted SCI recovery.

### 3.3. Exercise Training Affects the Electrophysiological Properties of Motor Neurons via Piezo1

Exercise training may improve post-SCI electrophysiological function by regulating Piezo channels. Studies indicate that the motor evoked potential (MEP) amplitude in the abductor pollicis muscle is significantly lower in SCI patients compared to healthy individuals, and exercise training can significantly increase both the amplitude and size of their MEPs [95, 96]. This phenomenon signifies that exercise training improves motor function by enhancing neural excitability and synaptic plasticity in motor neurons. The potential mechanisms by which exercise training improves nerve conduction and excitability are complex and likely multifactorial. The regulation of Piezo1 channel dynamics by adhesion molecules and their impact on synaptic stability is one potential pathway worthy of exploration [68, 70, 71]. However, neurons often exhibit pathological hyperexcitability after SCI, such as abnormally increased ion channel opening frequency [97]. Exercise training can effectively suppress such overexcitation: in SCI models, running training reduces the spontaneous firing frequency of deep dorsal horn interneurons [98]. The mechanism may be related to BDNF/TrkB pathway activation—by upregulating KCC2 and GABA expression, it restores neuronal chloride ion homeostasis to suppress abnormal excitation [99]. Notably, tFUS modulates MEP amplitude by activating Piezo1 channels on GABAergic neurons, mediating Ca^2+^ influx, and thereby enhancing GABAergic inhibitory transmission [100]. Exercise training may regulate excitability in a similarly intensity- and time-dependent manner. Furthermore, due to the complementary stimulus responsiveness of Piezo1 and Piezo2 [48], different exercise intensities may produce differential effects: high-intensity training may generally suppress the excitability of all motor neurons, while low-intensity training selectively suppresses only repetitively activated neurons [101].

## 4. Discussion

Piezo channels play a key potential mediating role in exercise-promoted recovery from SCI. Based on current evidence, Piezo channels may collectively exert neuromodulatory and reparative effects by mediating the multifaceted influences of exercise on CBF, the expression and signaling of neurotrophic factors, the function of adhesion molecules, the mechanical properties of the extracellular matrix, neuroendocrine responses, and neuronal electrophysiological properties (such as membrane potential and excitability).

Beyond the nervous system, the function of Piezo1 in the immune system is also exercise-related. High-intensity exercise or resistance training elevates blood pressure, increasing fluid shear stress (FSS). FSS activates the mechanosensitive ion channel Piezo1, mediating calcium signaling, which subsequently promotes T-cell activation and inflammatory cytokine secretion [102]. In the experimental autoimmune encephalomyelitis model, Piezo1 was shown to suppress the expansion and/or function of regulatory T cells (Tregs) by limiting the TGF-β signaling pathway [103]. However, another study revealed that in B cells, Piezo1 selectively promotes TGF-β1-dependent IgA class switching and antibody production by enhancing TGF-β1-induced Smad3 phosphorylation [104]. This suggests that mechanical force activation of Piezo1 may influence Treg differentiation or function in a cell-type-specific manner by regulating the TGF-β signaling pathway. Notably, the immunosuppressive properties of Tregs have been reported to mitigate inflammatory responses and promote functional recovery after SCI [105]. Piezo1 in the motor system is also activated in response to mechanical stimuli. Exercise can counteract disuse-induced muscle atrophy and promote muscle hypertrophy and bone remodeling [106–108]. Mechanical stretching applied to mouse extensor digitorum longus and gastrocnemius muscles activates Piezo1, promoting Ca^2+^ influx, and enhancing the fusion of muscle stem cells to form multinucleated cells or myofibers [109]. Furthermore, mechanical stimulation can also influence bone formation and differentiation via Piezo1 [110]. On the other hand, studies found that sensory neurons suppress the thermogenic activity of brown and beige adipose tissue through the mechanoreceptor Piezo2, preventing excessive energy expenditure and maintaining metabolic homeostasis [111]. This mechanism shares similarities with the effects of exercise intensity: the high activation threshold of Piezo2 may require high-intensity exercise to be triggered. Given that high-intensity exercise primarily relies on glycogen for energy, while moderate-to-low intensity exercise predominantly utilizes fat, this mechanism suggests Piezo2 may be a potential molecular basis for distinguishing the metabolic effects of different exercise intensities. Due to space limitations, this article does not delve into the potential roles of Piezo channels in other systems such as cardiovascular, respiratory, and digestive systems in response to exercise training.

Regulation of Piezo channels still carries potential risks. It has been reported that exercise training equally improves cardiovascular risk factors regardless of the initial endothelial function status, and endothelial function levels do not predict the magnitude of improvement [112]. However, under abnormal mechanical stimuli (such as turbulent blood flow or hypertension), excessive activation of Piezo1 can trigger inflammatory responses, disrupt the endothelial barrier, and abnormally activate the NF-κB pathway, thereby promoting atherosclerosis, thrombosis, and vascular dysfunction, increasing cardiovascular burden [113]. Simultaneously, the absence of Piezo2 in baroreceptor neurons impairs blood pressure regulation, leading to increased blood pressure variability, loss of reflexive heart rate adjustment, a predisposition to hypertension, and causing an imbalance in cardiovascular homeostasis [114]. Theoretically, Piezo2 hyperactivity might cause baroreflex hypersensitivity, leading to persistent hypotension and bradycardia, although this hypothesis was not directly tested in this study. Furthermore, the human body develops heat adaptation following high-intensity and high-temperature exercise [115]. This may also be related to Piezo2's involvement in heart rate and metabolic activity [111, 114]. Therefore, regulating the Piezo family through exercise still carries risks. Blood biomarker testing is a physiological monitoring method that can be used to assess physical status during exercise training [116]. Future research could explore whether circulating Piezo channel-related proteins or active products could serve as novel biomarkers for evaluating cardiovascular risk or exercise adaptation status.

Significant differences in the expression or function of Piezo family proteins also exist across populations, a factor that cannot be overlooked. Currently, these variant characteristics have primarily been observed in the blood and motor systems of individuals of African descent. Notably, exercise training of varying intensities may produce differential physiological effects across different populations [117].

## 5. Conclusions and Limitations

In summary, mechanosensitive ion channels Piezo represent a highly valuable research direction for mediating exercise interventions in promoting recovery from SCI. Based on current literature, we propose the core hypothesis: Piezo channels sense and transduce mechanical stimuli generated by exercise, potentially promoting structural remodeling and functional recovery of the cerebral cortex and spinal cord following SCI through the collective regulation of multiple pathways. These pathways include CBF, neurotrophic factor signaling, mechanical properties of the extracellular matrix, neuronal electrophysiological activity, glial cell functions, and immune responses. Specifically, Piezo channels may mediate the positive effects of exercise training on neuroregeneration, synaptic plasticity, axonal growth, and neuroprotection, and could potentially facilitate the reconstruction of damaged neural circuits.

However, this field still faces numerous key challenges and limitations. First, there is a critical need for mechanistic validation: this hypothesis primarily relies on non-SCI models or correlative studies, and direct causal evidence demonstrating Piezo channels specifically mediate exercise recovery effects in SCI animal models (e.g., using conditional knockout/overexpression combined with exercise intervention) remains scarce; exploration of underlying mechanisms in SCI animal models more closely resembling clinical scenarios (e.g., large animals and chronic injury models) is urgently needed. Second, a clinical translation gap exists: there is currently a lack of direct evidence from human clinical trials confirming the safety and efficacy of exercise strategies targeting Piezo channels in SCI patients; future research must focus on translating basic findings into assessable clinical interventions and rigorously validating them. Third, a void exists in parameter optimization: systematic research is still required to determine how to precisely optimize exercise parameters, such as type, intensity, frequency, and duration, to most effectively and safely regulate Piezo channel activity and maximize neuroreparative benefits. Fourth, cross-system interactions require consideration: while this review focused on the nervous system, Piezo channel regulation in other systems (e.g., cardiovascular, immune, and metabolic) by exercise training and their indirect impact on neural repair remain underexplored, necessitating future integrated cross-system studies. Fifth, model limitations persist: some supporting evidence (e.g., in vitro mechanical stretch studies) struggles to fully replicate the complex biomechanical and biological microenvironment of the spinal cord in vivo, warranting caution when extrapolating conclusions; developing more advanced in vivo biomechanical and molecular imaging technologies is needed to overcome this limitation. Addressing these challenges will establish a solid foundation for precision Piezo channel-based exercise rehabilitation strategies.

## Figures and Tables

**Table 1:** Human-Specific PIEZO1 Polymorphisms.

Variant locus	Associated phenotype/disease	Potential cause of variation	Functional impact/mechanism	Reference
rs62048221	Calcaneal bone mineral density	Mechanical loading during weight-bearing activities (standing, walking, and exercise)	Modulates activity of cis-regulatory elements, influencing PIEZO1 expression levels and subsequently affecting bone density	[34]

R1527H/ g.16-88737727-C-G	Primary open-angle glaucoma	Intraocular pressure (IOP)	Variant may reduce mechanosensitivity, potentially mitigating IOP-induced damage to the optic nerve	[35]

chr16:88716656-G-TT	HbA1c assay interference (South Asian populations)	Higher prevalence in South Asian populations; very rare in other ancestries	Artificially lowers reported HbA1c values, potentially delaying diabetes diagnosis	[36]

E756del(rs572934641)	Red blood cell	Malaria resistance	Confers protection by disrupting virulence protein trafficking in *Plasmodium*	[37]

E756del	Glaucoma	IOP	While highly prevalent in African ancestry populations, E756del showed no significant association with the studied glaucoma phenotype	[38]

rs2911463	Lower extremity deep vein thrombosis	Prolonged standing or walking speed	Potential pathogenic variant	[39]

rs202127176	Joint replacement surgery (arthritis)	Surgery	Potential role in response to structural joint destruction	[40]

E756del	Tendon properties	Adaptation to physical demands	Increases tendon stiffness and mechanical resilience	[41]

rs4782432 rs1061238 rs8043924 rs9928479	Chronic venous insufficiency (CVI)	Unknown genetic causes	Potential pathogenic variants	[42]

## Data Availability

As this article is a narrative review, no data were used or analyzed in the study.
